# MotifCombinator: a web-based tool to search for combinations of cis-regulatory motifs

**DOI:** 10.1186/1471-2105-8-100

**Published:** 2007-03-22

**Authors:** Mamoru Kato, Tatsuhiko Tsunoda

**Affiliations:** 1 Laboratory for Medical Informatics, SNP Research Center, RIKEN, Yokohama, Japan

## Abstract

**Background:**

A combination of multiple types of transcription factors and cis-regulatory elements is often required for gene expression in eukaryotes, and the combinatorial regulation confers specific gene expression to tissues or environments. To reveal the combinatorial regulation, computational methods are developed that efficiently infer combinations of cis-regulatory motifs that are important for gene expression as measured by DNA microarrays. One promising type of computational method is to utilize regression analysis between expression levels and scores of motifs in input sequences. This type takes full advantage of information on expression levels because it does not require that the expression level of each gene be dichotomized according to whether or not it reaches a certain threshold level. However, there is no web-based tool that employs regression methods to systematically search for motif combinations and that practically handles combinations of more than two or three motifs.

**Results:**

We here introduced *MotifCombinator*, an online tool with a user-friendly interface, to systematically search for combinations composed of any number of motifs based on regression methods. The tool utilizes well-known regression methods (the multivariate linear regression, the multivariate adaptive regression spline or MARS, and the multivariate logistic regression method) for this purpose, and uses the genetic algorithm to search for combinations composed of any desired number of motifs. The visualization systems in this tool help users to intuitively grasp the process of the combination search, and the backup system allows users to easily stop and restart calculations that are expected to require large computational time. This tool also provides preparatory steps needed for systematic combination search – *i.e*., selecting single motifs to constitute combinations and cutting out redundant similar motifs based on clustering analysis.

**Conclusion:**

*MotifCombinator *helps users to systematically search for motif combinations that play an important role in gene expression as measured by microarrays.

## Background

Gene expression in eukaryotes is controlled by combinatorial regulation of transcription factors and cis-regulatory elements. Many types of transcription factors are bound to their respective regulatory DNA elements, and the interactions between the factors and elements control the gene expression. Molecular experiments can identify several binding sites for selected transcription factors, but they are too laborious and time-consuming to be applied to large-scale studies. Computational methods are thus required for processing genomic data to reveal the combinatorial regulation on a genomic scale.

Recently, some computational methods have been developed to detect combinations of patterns (motifs) of cis-regulatory elements. They process data of upstream sequences selected from genomic sequences, as well as data of either expression levels measured by DNA microarrays or binding information given by ChIP-on-chip arrays [1-3]. One widely used type of computational method [4-6] employs genes that are co-expressed at a certain threshold level. Methods of this type first enumerate possible combinations of single motifs and then select significant combinations that specifically appear in upstream sequences of co-expressed genes [1,4,7]. Alternatively, such methods search directly for a pattern of several closely spaced motifs in the upstream sequences [5,6,8,9].

Another type of computational method is based on regression analysis between expression levels and motif scores (occurrence frequencies or weight matrix scores) in input sequences [10-12]. This type calculates a matching score of a motif (or motif combination) along each of the upstream sequences, for which the expression levels of the corresponding genes are measured by microarrays. It then takes regression between the motif scores in the upstream sequences and the expression levels of the corresponding genes to calculate the goodness-of-fit of the regression. This goodness-of-fit is obtained for each of the possible motifs (or motif combinations), and then motifs with the best fit are selected. These procedures are interpretable under the simple assumption that the occurrence frequencies or the weight matrix scores, which approximately correlate with the binding energy of the transcription factors to DNA elements [13], in upstream sequences influence the levels of gene expression [14], and that the scores of genuine motifs or motif combinations must explain much of the variation of expression levels. Well-known regression methods used for this purpose are the (multivariate) linear regression method [10,11] and the multivariate adaptive regression spline (MARS) method [12]. The former method assumes a linear function between the motif scores and expression levels, and the latter method has hockey-stick functions as basis functions, though it does not explicitly assume a particular function because it is a non-parametric method. This type of computational method can take full advantage of the information about expression levels, since it does not compulsively dichotomize expression levels by a threshold into a binary code to indicate whether or not a gene is expressed.

An integrated web tool (RgS-Miner [15]) to search for motif combinations has already been developed based on the widely used type of method above (searching for motif combinations specifically appearing in upstream sequences of co-expressed genes); however, there is no integrated web tool that employs regression methods to systematically search for motif combinations. Moreover, RgS-Miner and the previous regression methods practically handle combinations composed of only two or three motifs; however, more than two or three motifs can be involved in combinatorial regulation in higher organisms [16,17]. Hence, this case needs to be addressed.

We therefore developed *MotifCombinator*, a web-based tool that uses regression methods to systematically search for combinations of regulatory motifs. This tool is equipped with two kinds of regression methods, the multivariate linear regression and MARS methods, and it also includes logistic regression, which is a regression method for regulatory motifs that uses co-expressed genes [16]. It also employs the genetic algorithm to search for combinations composed of any desired number of motifs. For systematic combination search, *MotifCombinator *includes a series of procedures such as determining single motifs that will constitute motif combinations, filtering out redundant single motifs that resemble each other, and calculating the goodness-of-fit for possible combinations. As with the integrated tool RgS-Miner [15], these systematic procedures are realized in interactive multistage pipelines with a simple screen layout that can be easily used by experimental biologists. *MotifCombinator *will thus help users to find combinations of regulatory motifs that are important for combinatorial regulation.

## Implementation

### Multi-step pipelines

For the systematic search for motif combinations,* MotifCombinator *uses a four-step pipeline structure. The four steps are 1) uploading a data set of upstream sequences and gene expressions; 2) determining single motifs that will be used to constitute possible motif-combinations; 3) cutting out redundant or irrelevant single motifs; and 4) searching possible combinations composed of the filtered motifs. We will introduce the framework here; supplemental details on how to use the tool are also provided online.

### Preparatory steps

In the first step, users upload a data set consisting of both upstream sequences and gene expressions. Users can upload such a data set through files or by using a copy-and-paste function. Data of upstream sequences should be formatted in the FASTA format, and IDs of the upstream sequences should be indicated following a greater-than (">") symbol in the format. The gene expression data should consist of two columns, a column of IDs of gene expressions, and a column of expression levels or expression binaries. The expression levels are typically log ratios of expression levels for genes that are measured by microarrays, and will be subsequently processed by the linear regression or MARS. The expression binaries are 1 or 0, which indicate whether or not a gene is expressed, and will be subsequently processed by the logistic regression. After uploading both types of data, *MotifCombinator *matches up IDs in the sequence data with IDs in the expression data to manage together the both types of data. Users can also use pre-calculated non-homologous upstream sequences in the human and mouse genomes as sequence data.

The second step is to determine single motifs that will be used later to constitute candidate motif-combinations. Motifs are represented by position frequency matrices. These single motifs can be determined through motif-finding tools, the JASPAR database [18], and users' stored motifs. Three motif-finding tools based on different strategies are available to find *de novo *single motifs from input sequences: MEME [19], which mainly utilizes the EM algorithm, AnnSpec [20], which mainly utilizes Gibbs sampling, and MDscan [21], which mainly utilizes the word enumeration strategy. Users can adjust several parameters for these tools on the web screen. The TRANSFAC (and its module TRANSCompel) [22] and JASPAR [18] databases are among well-established databases of transcription factor binding motifs, but currently, only freely-downloadable JASPAR is pre-installed and its motifs can be uploaded with a simple mouse-click. Users can also upload their own motifs. After the upload, users can confirm the matrices of uploaded motifs and can delete them if necessary.

**Figure 1:**
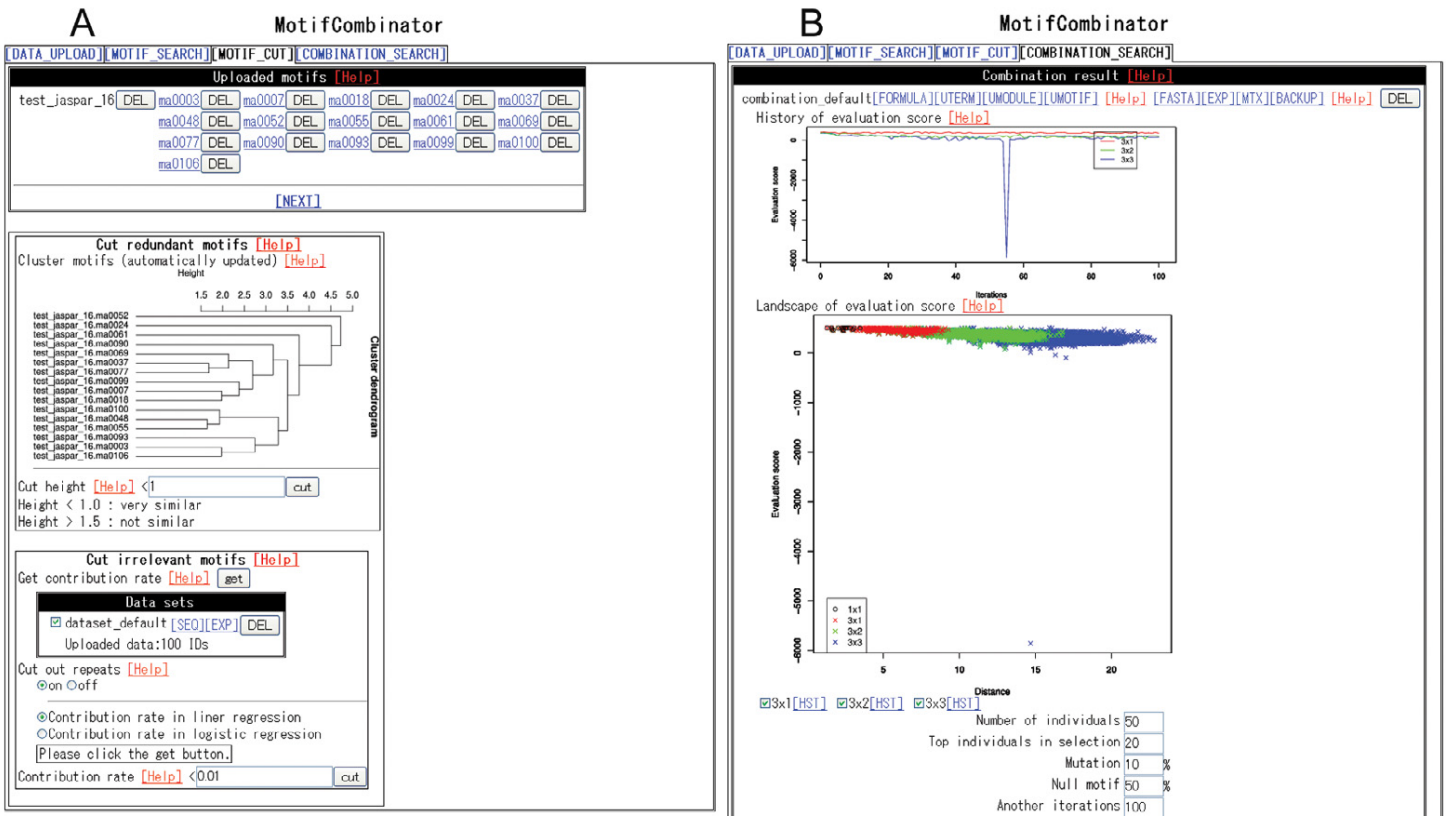
**View of *MotifCombinator***. (A) View at the step of motif cutting. The cluster figure shows the relationship between uploaded motifs. Users can cut out redundant similar motifs by simply clicking the button. (B) View at the step of combination search. Two figures are displayed on the web screen. The first figure ("History of evaluation score") shows the best goodness-of-fit value versus each of the iterations during the combination search. The dropped line in blue corresponds to finding the optimum motif combination here. The second figure ("Landscape of evaluation score") shows all goodness-of-fit values during the search versus the distance of all motif combinations from the reference motif (see the text). Different values of the distance are intended to represent different motif combinations. The point in blue near the bottom corresponds to the dropped line in the first figure and to the optimum motif combination here.

The third step (Figure 1A) is to cut out redundant or irrelevant single motifs. The single motifs used in combination searches sometimes include redundant – *i.e*., similar – motifs, which can result in combinations that are composed of many similar motifs. Such similar motifs also make the computational time unnecessarily long. In addition, single motifs for combination search may include irrelevant motifs that do not contribute to the expression levels and are likely not to contribute to them even if they are combined in a motif combination. Such irrelevant motifs also increase the computational time. Hence, it is necessary to cut out redundant or irrelevant single motifs at this stage before listing the possible combinations of single motifs at the next stage. For the first purpose, users can classify groups of similar motifs by clustering analysis and select one motif per cluster to obtain distinct motifs. Our tool uses the average Kullback-Leibler divergence calculated by MatCompare [23] (with the options of -D and -t 1e+30) as the distance between motifs to perform hierarchical clustering of the R language, and randomly selects one motif per cluster as a representative. For the second purpose, users can calculate how much variance in the input expression levels is explained by weight matrix scores of a single motif (in eq. 1 below when the number of modules is 1 and the number of motifs is also 1) in input sequences. For each single motif, the tool calculates the linear regression or the logistic regression between the motif scores in input sequences and input expression levels and then calculates the proportion of the variance of input expression levels explained by those expression levels that are predicted by the regression (the contribution rate, corresponding specifically to *R*^2 ^in the multivariate linear regression). A single motif that hardly contributes to the explanation of input expression levels may not contribute to the expression levels even if it is used to constitute a motif combination. Users can remove such a single motif.

### Combination search

At the fourth step (Figure 1B), users can search for motif combinations that are important for expression levels. *MotifCombinator *generates candidate motif combinations from motifs that are selected through the previous steps, or motifs that are selected at this step by users with checkboxes on the screen. For each of the motif combinations, the tool calculates the weight matrix scores (in eq. 1 below) of the combination in input sequences, and performs the regression between the scores and input expression levels to calculate the goodness-of-fit of the regression. It then selects motif combinations with the best fit. These procedures are iteratively performed through the genetic algorithm.

**Figure 2:**
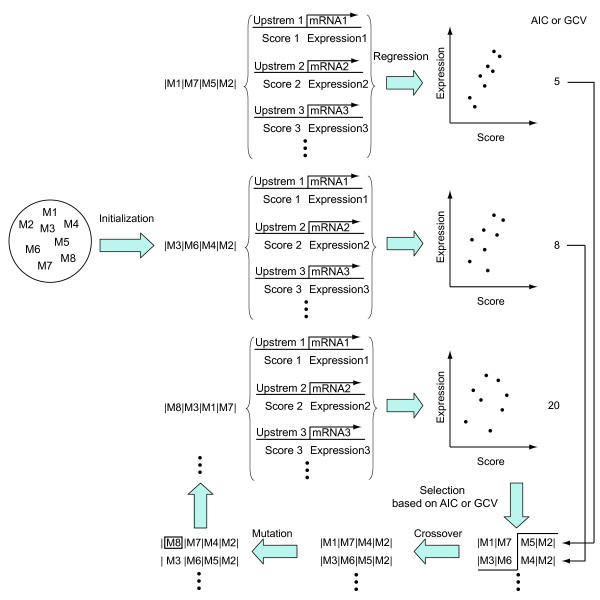
**Procedures of the genetic algorithm**. Ms (M1, M2, ...) indicate motifs. A series of motifs (*e.g*., |M1|M7|M5|M2|) is a motif combination composed of the motifs. At the initialization step, motifs are randomly selected to generate motif combinations. For each motif combination, matching scores are calculated from upstream sequences, and expression levels of mRNAs with the upstream sequences are obtained from microarray data. Regression between the scores and the expression levels is performed to obtain the goodness-of-fit (AIC or GCV), and motif combinations with the best goodness-of-fit values are selected to take crossover. Then mutation is performed to replace motifs with other motifs. These procedures are iteratively executed for updated motif combinations.

The genetic algorithm (Figure 2) consists of a coding system to express motif combinations and four procedures to handle the coded motif combinations. In the coding system, a motif combination, which in the language of the genetic algorithm is referred to as an *individual*, is coded in a series of names of uploaded motifs or the name NULL to indicate the absence of a motif. For example, the code |M1|M2|NULL|, where Ms denote motif names and NULL denotes not having a motif, represents a motif combination composed of motif M1 and M2. A more complex code is allowable. For example, the code |M1 M2|NULL M3|M4 M5| represents a motif combination that has a module composed of motif M1 and M2, a module composed of motif M3, and a module composed of motif M4 and M5. A module is a unit used in the scoring of a motif combination as described below. A user has to specify both the number of modules and the number of motifs that constitute modules on the web screen. A user can input values of greater than two; thus a user can search for combinations composed of more than two or three motifs, or rather, any number of motifs.

The first procedure in the genetic algorithm is initialization. Initial individuals (motif combinations) are randomly generated in the number that a user specifies. More specifically, motifs to constitute a motif combination are uniformly selected from all uploaded motifs at the probability of 0.5 (*i.e*., each of the uploaded motifs at the probability of 0.5 divided by the number of the uploaded motifs) and are selected from the null motif (NULL) at the probability of 0.5.

The second procedure is selection. For each of the motif combinations coded as described above, the matching scores of the motif combination are calculated from input sequences as follows:

Si,j=log⁡2{∑m∑wPr⁡(w from Mm)Pr⁡(w from M0)},     (eq. 1)
 MathType@MTEF@5@5@+=feaafiart1ev1aaatCvAUfKttLearuWrP9MDH5MBPbIqV92AaeXatLxBI9gBaebbnrfifHhDYfgasaacH8akY=wiFfYdH8Gipec8Eeeu0xXdbba9frFj0=OqFfea0dXdd9vqai=hGuQ8kuc9pgc9s8qqaq=dirpe0xb9q8qiLsFr0=vr0=vr0dc8meaabaqaciaacaGaaeqabaqabeGadaaakeaacqWGtbWudaWgaaWcbaGaemyAaKMaeiilaWIaemOAaOgabeaakiabg2da9iGbcYgaSjabc+gaVjabcEgaNnaaBaaaleaacqaIYaGmaeqaaOWaaiWaaeaadaaeqbqaamaaqafabaWaaSaaaeaacyGGqbaucqGGYbGCcqGGOaakcqWG3bWDcqqGGaaicqqGMbGzcqqGYbGCcqqGVbWBcqqGTbqBcqqGGaaicqWGnbqtdaWgaaWcbaGaemyBa0gabeaakiabcMcaPaqaaiGbccfaqjabckhaYjabcIcaOiabdEha3jabbccaGiabbAgaMjabbkhaYjabb+gaVjabb2gaTjabbccaGiabd2eannaaBaaaleaacqqGWaamaeqaaOGaeiykaKcaaaWcbaGaem4DaChabeqdcqGHris5aaWcbaGaemyBa0gabeqdcqGHris5aaGccaGL7bGaayzFaaGaeiilaWIaaCzcaiaaxMaadaqadaqaaiabbwgaLjabbghaXjabb6caUiabbccaGiabigdaXaGaayjkaiaawMcaaaaa@68FF@

where *i *and *j *denote the module and the sequence, *m *denotes any of the motifs belonging to the module *i*, *M*_*m *_is the probability matrix of the motif *m*, *M*_0 _is the third-order Markov model estimated from all input sequences, and *w *denotes a string of any of the sliding windows in the sequence *j*. When a user specifies the option of the number of motifs that constitute modules as one (*i.e*., no summation over *m*), this scoring is exactly the same as that used in the multivariate linear regression method [11]. Then, for the motif combination, the scores and input expression levels are regressed, and the goodness-of-fit of the regression is calculated. A user can select one of three regression methods: the multivariate linear regression [10,11], the multivariate adaptive regression spline (MARS) [12], or the multivariate logistic regression method [16]. The goodness-of-fit is Akaike's Information Criterion (AIC) for the linear/logistic regression, or the generalized cross-validation score (GCV) for MARS. By the goodness-of-fit, each motif combination (individual) is evaluated and only motif combinations with the best fit are selected in the number that a user specifies.

The third and fourth procedures are crossover and mutation. In the crossover, two different individuals are randomly chosen from the selected individuals with the best fit, and one-point crossover is performed between the codes of the two individuals to reproduce codes of two new individuals at the next generation (*e.g*., |M1|**|**|M2|M3| × |M4|**|**|M5|M6| → |M1|M5|M6| and |M4|M2|M3|). This operation is repeated until the number of new individuals at the next generation reaches that of the old individuals at the previous generation. At the fourth procedure, mutation is performed on codes of the new individuals. Here, mutation is random replacement of one motif with another at a site on the code of an individual (*e.g*., |M1|M2|M3| → |M4|M2|M3|). First, sites of motifs to be replaced are randomly selected across all individuals in the number of (the integer of) L*M, where *L *is the number of motifs in all individuals and *M *is the mutation rate specified by a user. Then old motifs at the selected sites are replaced with new motifs that are randomly selected as in the initialization step. By this procedure, one iteration loop is closed. The next iteration loop starts with the second procedure, selection, which in turn evaluates the new codes of motif combinations that are updated through crossover and mutation at the previous iteration. These iterations repeat for the number of times specified by the user.

On the web screen, users can obtain results about motif combinations with the best fit. The motif combinations listed there are the best ones throughout all iterations. By clicking the FORMULA button, users can see an estimated regression formula, together with the constituent motifs, regression coefficients, evaluation score (AIC or GCV), and contribution rate (the proportion of the variance of input expression levels explained by those expression levels that are predicted by the regression with the scores of a motif combination). By clicking the UMOTIF button, users can see motifs that constitute a motif combination. By referring to the two figures on the screen, users can intuitively grasp how the combination search has proceeded and thereby can get hints to evaluate the results. One figure on the screen shows the history of the best goodness-of-fit values during the combination search, *i.e*., the best goodness-of-fit value at the selection step versus each of the iterations during the search. If users observe, for example, that the values are steadily falling, in other words, getting better according to the iterations, this suggests that users should not stop the calculation but continue. The other figure on the screen shows a brief landscape of the goodness-of-fit values during the search, *i.e*., the values of all individuals in all iterations versus a metric whose different values are intended to represent different motif combinations. The metric is the sum of the distances of constituent motifs from the reference motif, of which the length is 10 and the probability matrix is composed of 0.25 for each nucleotide base. The distance is measured by the average Kullback-Leibler divergence between a constituent motif and the reference motif, and is calculated by MatCompare [23] (with the same options as above). If users observe, for example, that a point of the goodness-of-fit value is alone positioned extremely low in the y-axis (meaning good) and isolated far from all other points that are scattered thoroughly across the x-axis, this may suggest that the value is worthwhile and the motif combination with this value may be close to the optimal solution.

Finally, our tool has a backup system for re-calculation later, since the combination search takes a long time when the sizes of the parameters are large. Users can dump an archive file needed for re-calculation by simply clicking the BACKUP button. Then, they can shut down the computer. Users can easily restart the calculation by uploading the archive file on the web screen.

In summary, the characteristic points at the combination search are as follows.

• Users can search for combinations composed of any number of motifs, using the genetic algorithm.

• Users can use the three types of regression methods (the multivariate linear regression, MARS, and the multivariate logistic regression) to find motif combinations that well explain the variations of expression levels.

• Users can intuitively understand how the search has proceeded by the two visualization systems, which show a history and a brief landscape of the goodness-of-fit scores during the search.

• Users can easily store all records by the backup system, and even after shutting down the computer, they can easily restart the search by just uploading the backup file on the web screen.

## Results and discussion

### Tests by simulated data

Using simulated data sets, we tested whether the tool can correctly recover motif combinations composed of given motifs from many irrelevant motifs. The simulated data sets consisted of simulated upstream sequences and simulated expression levels. We generated the simulated upstream sequences in 2000 bps by the third-order Markov model estimated from the human genome. Into the upstream sequences, we randomly planted modules with motifs neighboring within 100 bps of each other, following the Poisson distribution (with a mean of one and a maximum number of five) for the number of modules in each upstream sequence, and following the probability matrices of motifs to plant strings of the motifs into each upstream sequence. We generated ten different sets of such simulated upstream sequences, into which we respectively planted ten different modules.

We generated two types of simulated expression levels. One was generated by the linear combination of the scores of modules as follows:

∑iaixig+C+kεg,     (eq. 2)
 MathType@MTEF@5@5@+=feaafiart1ev1aaatCvAUfKttLearuWrP9MDH5MBPbIqV92AaeXatLxBI9gBaebbnrfifHhDYfgasaacH8akY=wiFfYdH8Gipec8Eeeu0xXdbba9frFj0=OqFfea0dXdd9vqai=hGuQ8kuc9pgc9s8qqaq=dirpe0xb9q8qiLsFr0=vr0=vr0dc8meaabaqaciaacaGaaeqabaqabeGadaaakeaadaaeqbqaaiabdggaHnaaBaaaleaacqWGPbqAaeqaaOGaemiEaG3aaSbaaSqaaiabdMgaPjabdEgaNbqabaGccqGHRaWkcqWGdbWqcqGHRaWkcqWGRbWAiiGacqWF1oqzdaWgaaWcbaGaem4zaCgabeaaaeaacqWGPbqAaeqaniabggHiLdGccqGGSaalcaWLjaGaaCzcamaabmaabaGaeeyzauMaeeyCaeNaeeOla4IaeeiiaaIaeGOmaidacaGLOaGaayzkaaaaaa@47CC@

where *i *and *g *are the indices of a module and a gene, respectively, *x *is the score (in eq. 1) of a module, *C *and *k *are constants, and ε is a *N *(0,1) Gaussian noise. *C *was adjusted for the mean of expression levels across genes to be zero, and *k *was adjusted for the standard deviation of noises to be a certain percent of the standard deviation of expression levels across genes. We used this type of simulated expression level for the test of the linear regression method. The other simulated expression levels were generated by the two-order interaction terms between different modules in addition to the linear combination [12] as follows:

∑iaixig+∑i<jbijxigxjg+C+kεg.     (eq. 3)
 MathType@MTEF@5@5@+=feaafiart1ev1aaatCvAUfKttLearuWrP9MDH5MBPbIqV92AaeXatLxBI9gBaebbnrfifHhDYfgasaacH8akY=wiFfYdH8Gipec8Eeeu0xXdbba9frFj0=OqFfea0dXdd9vqai=hGuQ8kuc9pgc9s8qqaq=dirpe0xb9q8qiLsFr0=vr0=vr0dc8meaabaqaciaacaGaaeqabaqabeGadaaakeaadaaeqbqaaiabdggaHnaaBaaaleaacqWGPbqAaeqaaOGaemiEaG3aaSbaaSqaaiabdMgaPjabdEgaNbqabaGccqGHRaWkdaaeqbqaaiabdkgaInaaBaaaleaacqWGPbqAcqWGQbGAaeqaaOGaemiEaG3aaSbaaSqaaiabdMgaPjabdEgaNbqabaGccqWG4baEdaWgaaWcbaGaemOAaOMaem4zaCgabeaaaeaacqWGPbqAcqGH8aapcqWGQbGAaeqaniabggHiLdGccqGHRaWkcqWGdbWqcqGHRaWkcqWGRbWAiiGacqWF1oqzdaWgaaWcbaGaem4zaCgabeaaaeaacqWGPbqAaeqaniabggHiLdGccqGGUaGlcaWLjaGaaCzcamaabmaabaGaeeyzauMaeeyCaeNaeeOla4IaeeiiaaIaeG4mamdacaGLOaGaayzkaaaaaa@5B87@

We used this type of simulated expression level for the test of MARS. We added the Gaussian noises at 0%, 20%, 40%, 60%, and 80% of the standard deviation of expression levels across genes.

For these data sets (10 sets of modules × 2 types of expression levels × 5 degrees of noises), we tested whether *MotifCombinator *can correctly recover motif combinations of planted modules from all JASPAR [18] motifs (111 motifs). For all JASPAR motifs, we first performed the step of motif cutting based on the clustering analysis at a cut height of 1. Then at the step of combination search, we set the options as follows: number of individuals = 50, top individuals in selection = 20, mutation = 10%, and iterations = 500. We set module × motif according to the modules used (*e.g*., when three modules with two motifs were used, we set 3 × 2) and set the regression type to be the linear regression and MARS for the two types of expression levels in eq. 2 and eq. 3, respectively. Using these settings, we executed the search and obtained the results. For each data set, we took up the top ten motif combinations (by the UMOTIF button) that were evaluated and recovered by the tool. We calculated how well the tool recovered a motif combination, based on the hypergeometric test:

p(t)={(Tt)×(K−Tk−t)}/(Kk),
 MathType@MTEF@5@5@+=feaafiart1ev1aaatCvAUfKttLearuWrP9MDH5MBPbIqV92AaeXatLxBI9gBaebbnrfifHhDYfgasaacH8akY=wiFfYdH8Gipec8Eeeu0xXdbba9frFj0=OqFfea0dXdd9vqai=hGuQ8kuc9pgc9s8qqaq=dirpe0xb9q8qiLsFr0=vr0=vr0dc8meaabaqaciaacaGaaeqabaqabeGadaaakeaacqWGWbaCcqGGOaakcqWG0baDcqGGPaqkcqGH9aqpdaWcgaqaamaacmaabaWaaeWaaeaafaqabeGabaaabaGaemivaqfabaGaemiDaqhaaaGaayjkaiaawMcaaiabgEna0oaabmaabaqbaeqabiqaaaqaaiabdUealjabgkHiTiabdsfaubqaaiabdUgaRjabgkHiTiabdsha0baaaiaawIcacaGLPaaaaiaawUhacaGL9baaaeaadaqadaqaauaabeqaceaaaeaacqWGlbWsaeaacqWGRbWAaaaacaGLOaGaayzkaaaaaiabcYcaSaaa@4858@

P=1−∑i=0t−1p(i),
 MathType@MTEF@5@5@+=feaafiart1ev1aaatCvAUfKttLearuWrP9MDH5MBPbIqV92AaeXatLxBI9gBaebbnrfifHhDYfgasaacH8akY=wiFfYdH8Gipec8Eeeu0xXdbba9frFj0=OqFfea0dXdd9vqai=hGuQ8kuc9pgc9s8qqaq=dirpe0xb9q8qiLsFr0=vr0=vr0dc8meaabaqaciaacaGaaeqabaqabeGadaaakeaacqWGqbaucqGH9aqpcqaIXaqmcqGHsisldaaeWbqaaiabdchaWjabcIcaOiabdMgaPjabcMcaPaWcbaGaemyAaKMaeyypa0JaeGimaadabaGaemiDaqNaeyOeI0IaeGymaedaniabggHiLdGccqGGSaalaaa@3EF7@

where *K *is the number of all motifs, *k *is the number of motifs (say, true motifs) that constitute a motif combination we planted, *T *is the number of all motifs in a motif combination recovered by the tool, and *t *is the number of true motifs included in the motif combination recovered by the tool. We calculated the *P *value for each of the top ten motif combinations in each data set, and kept two types of *P *values: the best *P *value among the top ten and the (log) averaged *P *value across the top ten in each data set. Then we averaged both *P *values across all data sets. Table 1 shows that, in the case of the averaged *P *values across the top ten ("Average"), the values were always under 0.01 (1% significance level) in all noise and regression types, though the values got worse according to the increase of noise. The best *P *values ("Best") were always under roughly 10^-4 ^(< 2.0 × 10^-4^). The tool significantly recovered motif combinations that were exactly or approximately the same as those we planted.

**Table 1:** Evaluation of the tool by simulated data sets

Expression type	Noise (%)	Log_10_(*P*)	C.R.	Log_10_(*P*)	C.R.
		
		Average		Best	
Linear	0	-2.9	0.83	-4.8	0.89
	20	-2.9	0.80	-4.9	0.85
	40	-2.7	0.73	-4.5	0.78
	60	-2.7	0.63	-4.3	0.68
	80	-2.2	0.52	-3.7	0.55

Quadratic	0	-3.9	0.82	-5.8	0.88
	20	-3.7	0.78	-5.8	0.85
	40	-3.6	0.73	-5.5	0.77
	60	-3.0	0.63	-4.7	0.69
	80	-2.9	0.57	-4.6	0.58

We tested if the tool can correctly recover planted motif combinations, using simulated data sets. We evaluated the results based on *P *values of the hypergeometric test (see the text). "Linear" and "Quadratic" indicate types of simulated expression levels given by eq. 2 and eq. 3, respectively, and "Noise" indicates the degree of noises added into the simulated expression levels. "Average" and "Best" correspond to the cases of the *P *value (logarithmically) averaged across the top 10 motif combinations and the best *P *value among the top 10, respectively (see the text). "Log_10_(*P*)" indicates log_10 _of the *P *values. "C.R." indicates the contribution rate, which is the proportion of the variance of input expression levels explained by the scores of a motif combination. "Log_10_(*P*)" and "C.R." values in each row are averaged across ten data sets.

### Tests by muscle-specific transcripts

We tested the tool using real data sets, which consisted of upstream sequences and expression levels of muscle-specific transcripts. We obtained skeletal muscle-specific transcripts and their expression levels from a data file (Additional data file 5) included in a study of an extensive microarray survey of gene expression in normal human tissues [24]. That study measured the expression levels in two tissues related to skeletal muscle: abdominal muscle and right calf muscle. For the upstream sequences, we used genome sequences in the human NCBI build 35 and obtained sequences 2000-bps upstream (without overlapping other transcripts) from the muscle-specific transcripts. A set of 51 muscle-specific transcripts was uploaded into the tool. We also prepared position weight matrices of motifs (MEF2, MYF, SP1, SRF, and TEF) known to be involved in muscle-specific expression [16] using the JASPAR database [18], and uploaded them.

We tested whether the tool could correctly select motif combinations composed of these motifs from among all JASPAR motifs (111 motifs). We first performed the step of motif cutting based on the clustering analysis at a cut height of 1.5. Then, in the step of combination search, we set the options as follows: number of individuals = 50, top individuals in selection = 20, mutation = 10%, and iterations = 500. We set module × motif to 1 × 5 and regression type to MARS. Among a dozen of the top motif combinations that were evaluated and selected by the tool, three (MEF2, MYF, and SRF) were found out of the five muscle-related motifs in the abdominal-muscle data set and three (MEF2, SP1, and SRF) were found in the right calf-muscle data set. For each motif combination, we evaluated the results by the hypergeometric test described above. Table 2 shows that, in the abdominal-muscle data set, the best *P *value was under 0.01 and the combinations with the best *P *value included MEF2 and SRF, and MYF and SRF. In the right calf-muscle data set, the best *P *value was roughly 10^-4 ^(1.7 × 10^-4^) and the combination with the best *P *value included MEF2, SP1, and SRF.

**Table 2:** Evaluation of the tool by muscle-specific transcripts

Examined tissue	Best log_10_(*P*)	C.R.	Selected muscle motifs
Muscle, abdominal	-2.02	0.43	MEF2, SRF
	-2.02	0.42	MYF, SRF
Muscle, right calf	-3.75	0.52	MEF2, SP1, SRF

We used upstream sequences and expression levels of muscle-specific transcripts [24] to see if the tool can select a motif combination composed of motifs (MEF2, MYF, SP1, SRF, and TEF) involved in muscle-specific expression from all JASPAR [18] motifs. We employed MARS as the regression method. We evaluated the results based on *P *values of the hypergeometric test (see the text). "Examined tissue" indicates tissues that the study [24] examined as tissues of skeletal muscle. We list here results on a motif combination(s) with the best *P *value among a dozen of the top motif combinations selected by the tool. "Log_10_(*P*)" indicates log_10 _of the *P *values. "C.R." indicates the contribution rate, which is the proportion of the variance of input expression levels explained by the scores of a motif combination. "Selected muscle motifs" indicates muscle-related motifs that were included in the motif combination selected by the tool.

## Conclusion

Sequencing of genomic DNA deepens our understanding of DNA codes of protein products on genomic sequences in organisms; however, the codes that control gene expression or protein products are not well understood because of their complexity. Such complexity arises partly from the combinatorial regulation of cis-regulatory elements – many short DNA segments are combined to confer the ability to express genes specifically to tissues or environments. To understand the combinatorial regulation, it is necessary to develop a computational tool that efficiently searches possible combinations of patterns (motifs) of cis-regulatory elements to find combinations important for gene expression measured by, for example, microarrays.

We developed *MotifCombinator*, a web-based tool that searches for combinations composed of any desired number of motifs using the genetic algorithm and that employs well-known regression methods to find motif combinations that well explain the variations of expression levels directly – without dichotomizing expression levels into the "expressed" or "non-expressed" categories. This tool also has two visualization systems for intuitive evaluation of the search and has a backup system for continuing a long calculation. Convenient preparatory steps (selecting single motifs as "seeds" for combinations, cutting out redundant motifs) are also implemented for systematic search of the combinations. Using simulated data sets and muscle-specific transcripts, we tested the tool and found that the tool indeed recovered appropriate combinations of motifs for these data. Recently, information on known motif combinations has been increasing, as in TRANSCompel [22], which is a database of two (or more) closely-located binding sites on DNA sequences. This information can in principle be integrated into a system that handles combinations of any number of position frequency matrices, such as our tool, and the integration would increase the accuracy and convenience of the system. In conclusion, *MotifCombinator *will help users to efficiently search for motif combinations that are important for gene expression measured in genome-wide experiments.

## Availability and requirements

Project name: *MotifCombinator*

Project home page: 

Operating system(s): Platform independent (web-based, tested on Mozilla Firefox 1.5 and Internet Explorer 6.0)

Any restrictions to use by non-academics: None

## Abbreviations

- MARS: the multivariate adaptive regression spline

- AIC: Akaike's Information Criterion

- GCV: the generalized cross-validation score

- eq.: equation

## Authors' contributions

MK planned the research, performed the analyses, and wrote the manuscript. TT reviewed the manuscript. All authors read and approved the final manuscript.

## References

[B1] Kato M, Hata N, Banerjee N, Futcher B, Zhang MQ (2004). Identifying combinatorial regulation of transcription factors and binding motifs. Genome Biol.

[B2] Zhu Z, Shendure J, Church GM (2005). Discovering functional transcription-factor combinations in the human cell cycle. Genome Res.

[B3] Wang W, Cherry JM, Nochomovitz Y, Jolly E, Botstein D, Li H (2005). Inference of combinatorial regulation in yeast transcriptional networks: a case study of sporulation. Proc Natl Acad Sci U S A.

[B4] Aerts S, Van Loo P, Moreau Y, De Moor B (2004). A genetic algorithm for the detection of new cis-regulatory modules in sets of coregulated genes. Bioinformatics.

[B5] Gupta M, Liu JS (2005). De novo cis-regulatory module elicitation for eukaryotic genomes. Proc Natl Acad Sci U S A.

[B6] Zhou Q, Wong WH (2004). CisModule: de novo discovery of cis-regulatory modules by hierarchical mixture modeling. Proc Natl Acad Sci U S A.

[B7] Sudarsanam P, Pilpel Y, Church GM (2002). Genome-wide co-occurrence of promoter elements reveals a cis-regulatory cassette of rRNA transcription motifs in Saccharomyces cerevisiae. Genome Res.

[B8] GuhaThakurta D, Stormo GD (2001). Identifying target sites for cooperatively binding factors. Bioinformatics.

[B9] Liu X, Brutlag DL, Liu JS (2001). BioProspector: discovering conserved DNA motifs in upstream regulatory regions of co-expressed genes. Pac Symp Biocomput.

[B10] Roven C, Bussemaker HJ (2003). REDUCE: An online tool for inferring cis-regulatory elements and transcriptional module activities from microarray data. Nucleic Acids Res.

[B11] Conlon EM, Liu XS, Lieb JD, Liu JS (2003). Integrating regulatory motif discovery and genome-wide expression analysis. Proc Natl Acad Sci U S A.

[B12] Das D, Banerjee N, Zhang MQ (2004). Interacting models of cooperative gene regulation. Proc Natl Acad Sci U S A.

[B13] Stormo GD (2000). DNA binding sites: representation and discovery. Bioinformatics.

[B14] Bussemaker HJ, Li H, Siggia ED (2001). Regulatory element detection using correlation with expression. Nat Genet.

[B15] Huang HD, Horng JT, Sun YM, Tsou AP, Huang SL (2004). Identifying transcriptional regulatory sites in the human genome using an integrated system. Nucleic Acids Res.

[B16] Wasserman WW, Fickett JW (1998). Identification of regulatory regions which confer muscle-specific gene expression. J Mol Biol.

[B17] Berman BP, Nibu Y, Pfeiffer BD, Tomancak P, Celniker SE, Levine M, Rubin GM, Eisen MB (2002). Exploiting transcription factor binding site clustering to identify cis-regulatory modules involved in pattern formation in the Drosophila genome. Proc Natl Acad Sci U S A.

[B18] Sandelin A, Alkema W, Engstrom P, Wasserman WW, Lenhard B (2004). JASPAR: an open-access database for eukaryotic transcription factor binding profiles. Nucleic Acids Res.

[B19] Bailey TL, Elkan C (1994). Fitting a mixture model by expectation maximization to discover motifs in biopolymers. Proc Int Conf Intell Syst Mol Biol.

[B20] Workman CT, Stormo GD (2000). ANN-Spec: a method for discovering transcription factor binding sites with improved specificity. Pac Symp Biocomput.

[B21] Liu XS, Brutlag DL, Liu JS (2002). An algorithm for finding protein-DNA binding sites with applications to chromatin-immunoprecipitation microarray experiments. Nat Biotechnol.

[B22] Matys V, Kel-Margoulis OV, Fricke E, Liebich I, Land S, Barre-Dirrie A, Reuter I, Chekmenev D, Krull M, Hornischer K, Voss N, Stegmaier P, Lewicki-Potapov B, Saxel H, Kel AE, Wingender E (2006). TRANSFAC and its module TRANSCompel: transcriptional gene regulation in eukaryotes. Nucleic Acids Res.

[B23] Schones DE, Sumazin P, Zhang MQ (2005). Similarity of position frequency matrices for transcription factor binding sites. Bioinformatics.

[B24] Shyamsundar R, Kim YH, Higgins JP, Montgomery K, Jorden M, Sethuraman A, van de Rijn M, Botstein D, Brown PO, Pollack JR (2005). A DNA microarray survey of gene expression in normal human tissues. Genome Biol.

